# Cell therapy for retinal degenerative disorders: a systematic review and three-level meta-analysis

**DOI:** 10.1186/s12967-024-05016-x

**Published:** 2024-03-02

**Authors:** Alireza Soltani Khaboushan, Negar Ebadpour, Mohammad Mehdi Johari Moghadam, Zahra Rezaee, Abdol-Mohammad Kajbafzadeh, Masoumeh Majidi Zolbin

**Affiliations:** 1 Pediatric Urology and Regenerative Medicine Research Center, Children’s Medical Center, Gene, Cell and Tissue Research Institute, Tehran University of Medical Science, Tehran, Iran; 2 Students’ Scientific Research Center, Tehran University of Medical Sciences, Tehran, Iran; 3 Department of Ophthalmology & Vision Science, Tschannen Eye Institute, University of California, Davis, Sacramento, CA USA; 4 Department of Medical Genetics, Faculty of Medical Sciences, Tarbiat Modares University, Tehran, Iran

**Keywords:** Retinal Disease, MSCs, iPSCs, hESCs, Cell therapy

## Abstract

**Background:**

Retinal degenerative disorders (RDDs) cause vision loss by damaging retinal neurons and photoreceptors, affecting individuals of all ages. Cell-based therapy has emerged as an effective approach for the treatment of RDDs with promising results. This meta-analysis aims to comprehensively evaluate the efficacy of cell therapy in treating age-related macular degeneration (AMD), retinitis pigmentosa (RP), and Stargardt macular degeneration (SMD) as the most prevalent RDDs.

**Methods:**

PubMed, Scopus, Web of Science, and Embase were searched using keywords related to various retinal diseases and cell therapy treatments until November 25th, 2023. The studies’ quality was evaluated using the Joanna Briggs Institute’s (JBI) checklist for quasi-experimental studies. Visual acuity measured as LogMAR score was used as our main outcome. A three-level random-effect meta-analysis was used to explore the visual acuity in patients who received cell-based therapy. Heterogeneity among the included studies was evaluated using subgroup and sensitivity analyses. Moreover, meta-regression for the type of cells, year of publication, and mean age of participants were performed.

**Results:**

Overall, 8345 studies were retrieved by the search, and 39 met the eligibility criteria, out of which 18 studies with a total of 224 eyes were included in the meta-analysis. There were 12 studies conducted on AMD, 7 on SMD, and 2 on RP. Cell therapy for AMD showed significant improvement in LogMAR (*p* < 0.05). Also, cell therapy decreased the LogMAR score in SMD and RP (*p* < 0.01 and *p* < 0.0001, respectively). Across all conditions, no substantial publication bias was detected (p < 0.05).

**Conclusion:**

The findings of the study highlight that the application of cell therapy can enhance the visual acuity in AMD, SMD, and RP.

**Supplementary Information:**

The online version contains supplementary material available at 10.1186/s12967-024-05016-x.

## Background

A considerable portion of the global population suffers from visual impairment and even permanent vision loss due to a group of heterogeneous diseases collectively known as retinal degenerative disorders (RDDs). Age-related macular degeneration (AMD) is the most common form of retinal degenerative problem, with an approximate prevalence of 20 million in the United States and 196 million globally. These diseases are characterized by the progressive deterioration of the retina, leading to the loss of photoreceptor cells and subsequent vision loss. The incidence of vision loss caused by Retinal Detachment (RD) is on the rise [1–4]. The characteristic pathological manifestations of RDD involve the degeneration and demise of photoreceptors (rods and cones), retinal ganglion cells (RGCs), and retinal pigment epithelium (RPE) cells, which exhibit an inability to regenerate. RDD can manifest in various forms, such as AMD, retinitis pigmentosa (RP), and less well-known inherited retinal dystrophies like Stargardt macular degeneration (SMD) [5, 6].

Age-related macular degeneration is a prevalent eye disease affecting millions of people worldwide and is widely recognized as the primary factor contributing to permanent vision loss among adults aged 60 and above in the developed world. Two primary categories of this disease exist: neovascular (wet) and non-neovascular (dry). Dry AMD, including approximately 80% to 85% of patients, exhibits a better visual prognosis. At the same time, neovascular AMD impacts the residual 15% to 20% of cases and is responsible for 80% of severe vision loss [7].

AMD is characterized by pathological alterations in the macula and its adjacent vasculature, resulting in the progressive impairment of central vision. Retinal deposits, known as drusen, are a significant clinical hallmark observed in individuals with age-related macular degeneration. Dry AMD is the prevailing morphological subtype and has the potential to advance into the neovascular type [8].

Another important external retinal disease is retinitis pigmentosa (RP), which is a commonly hereditary and severe degenerative retinal disease characterized by the gradual loss of photoreceptor cells and atrophy of the RPE. In the early stages, nyctalopia occurs, followed by a gradual deterioration of visual acuity, resulting in loss of vision. Globally, there is an observed increase in the prevalence of early-onset RP variants, likely due to advancements in genetic screening techniques. This visual impairment typically becomes more apparent in individuals between the ages of 40 and 50. Despite progress in the therapeutic methods, there is still no approved effective treatment for RP [2, 9, 10].

Stargardt macular degeneration is an inherited ocular disorder that leads to a gradual loss of visual acuity, mostly impacting the macula. In the majority of individuals diagnosed with SMD, there is a progressive accumulation of lipofuscin (a fatty yellow pigment) within the cells located beneath the macula, damaging cells critical for clear central vision. Additionally, this disease causes nocturnal visual impairment, and certain patients may experience compromised color vision. The manifestation of symptoms is commonly observed throughout the later stages of infancy, extending into early adulthood, and exhibits a progressive deterioration as time progresses [11, 12].

Generally, these conditions carry significant effects on quality of life, such as heightened susceptibility to falls, depression, and a greater reliance on long-term care services. Additionally, visual impairments caused by RDD can vary in severity and progression based on their type, and the onset of these diseases usually ranges from congenital to late adulthood, therefore making them complex and challenging conditions to treat effectively [10].

Stem cells, characterized by their ability to undergo self-renewal and differentiation into specialized cell types, have garnered attention as a potential treatment for a range of pathological conditions such as degenerative retinal diseases. The retina is a highly favorable candidate for stem cell therapies due to its accessibility, innovative surgical techniques, limited diversity of cell types, compact organ size, and immune-privileged characteristics [13].

On the contrary, conventional therapeutic approaches aimed at addressing retinal degeneration have been ineffective in terms of restoring and regenerating the impaired retina. The application of stem cell-based therapy has emerged as a promising approach in the treatment of retinal degeneration, owing to its remarkable attributes such as self-renewal, multi-directional differentiation, neuroprotection, and immuno-regulation. Additionally, they have the ability to act as inhibitors of neuronal cell apoptosis and promote the release of neurotrophins. Hence, the objective of stem cell replacement therapy in these diseases is to generate new retinal cells from stem cells to substitute injured photoreceptor cells and outer nuclear layers [14, 15].

Various types of stem cells, including induced pluripotent stem cells (iPSCs), mesenchymal stem cells (MSCs), and retinal progenitor cells (RPCs), are presently under investigation in phase 1 and 2 clinical trials for retinal degenerative diseases (RDDs) such as age-related macular degeneration (AMD), inherited retinal dystrophies, and retinal vascular disorders. These stem cells may be sourced from embryonic origins, known as embryonic stem cells (ESCs), or from adult sources, known as adult stem cells (ASCs) [16, 17].

In addition, the wide array of current methods for evaluating ocular structure and function enables continuous monitoring and surveillance of stem cell activity, positioning retinal conditions as a prominent focus in stem cell-oriented clinical investigations [13]. This review specifically aims to comprehensively synthesize and evaluate the effectiveness of cell therapies in addressing AMD, SMD, and RP. We conduct a meta-analysis of published clinical trial data, with a concentrated focus on assessing the outcomes and efficacy of these therapeutic interventions.

## Methods

### Study protocol and search strategy

This study followed the PRISMA (Preferred Reporting Items for Systematic Reviews and Meta-Analyses) protocols for conducting a systematic review and meta-analysis. PubMed, Scopus, Web of Science, and Embase databases were searched using keywords of macular degeneration, retinal degeneration, stargardt's disease, macular dystrophy, retinitis pigmentosa, stem cell, regenerative medicine, cell therapy, extracellular matrix, and scaffold. A comprehensive list of keywords and search strategies for all databases is provided in Additional file 1. No restrictions were set on the search, and the search has been updated until November 25th, 2023. The protocol of the study has been registered in the PROSPERO (https://www.crd.york.ac.uk/prospero/display_record.php?RecordID=299200) with submission ID of CRD42022299200. A search for the gray literature was conducted using Google Scholar.

### Eligibility criteria and screening

The studies that used cell therapies for retinal degeneration were considered for this review. The inclusion criteria were clinical trials that used cell therapy as an intervention on patients with AMD, SMD, and RP, provided enough information on the details of the procedure, and assessed the visual acuity of the patients in the follow-up visits. The exclusion criteria were: (1) review, letter articles, and studies with non-original data, (2) animal studies, (3) in vitro studies, (4) studies that do not have a cell therapy intervention, and (5) lack of baseline assessment. Two authors (NE and ZR) independently screened retrieved studies based on the criteria using title and abstract. Two authors (NE and ZR) performed the full-text assessment independently based on the same criteria. Conflicts were resolved by consulting with the third reviewer (ASK).

### Data extraction

Two authors (NE and ZR) independently extracted data from all included studies. After extraction, the authors cross-checked their extracted data for any potential discrepancies. The extracted data was checked by the third author (ASK), and discrepancies were resolved. The following data were extracted from the studies: year of publication, first author’s name, type of study, type of retinal degeneration, inclusion and exclusion criteria of patients, sample size, demographic information (e.g., age and sex), source and type of applied cells, concentration of cells, procedure of application, and visual acuity. The missing data were retrieved by contacting the corresponding authors. In case data was presented in figures and plots, WebPlotDigitizer was used to extract it (https://apps.automeris.io/wpd/).

### Quality assessment

Assessment of the studies' risk of bias was conducted utilizing the Joanna Briggs Institute’s (JBI) checklist for quasi-experimental studies [18]. In this scale, each study is evaluated based on nine items, including assessment for cause and effect, participant comparison, intervention, control, pre and post-intervention outcome measure, follow-up, outcome measure comparison, reliability of outcome measure, and statistical analysis. The detailed questions are provided in Table 2 and in Additional file 2. Each item was graded as 1 (yes), 0 (no), or NA (Not Applicable or Unclear). Two authors (ASK and NE) used the JBI Scale to independently assess quality, resulting in a score from 0 to 9. Discrepancies and uncertainness regarding questions were resolved by the third reviewer (MMJM).

### Statistical analysis and data synthesis

Vision acuity in the LogMAR scale was collected as mean ± standard deviation (SD). In case median and interquartile range (IQR) were reported instead, they were converted to mean ± SD [19–21]. To homogenize the measures so they could be pooled, all visual acuity measures (ETDRS letter score and Snellen scale) were converted to LogMAR using the *eye* package in the R programming language [22]. Hedges’ g standardized mean difference and 95% confidence interval (CI) were used to calculate the effect size. Since there were multiple follow-ups, hence multiple effects from one study, a three-level meta-analysis using restricted maximum likelihood (REML) was used to prevent unit-of-analysis issues and handle the violation of independence assumption. To assess the goodness of the fit of the three-level model and compare it with the conventional model, we used log-likelihood-ratio tests. Funnel plot asymmetry and Egger’s test were used to assess the publication bias. If there is any publication bias, we will employ the Trim-and-fill method to address it. In the AMD meta-analysis, a subgroup analysis was performed based on whether the type of disease was wet or dry to investigate the source of heterogeneity. Moreover, meta-regression was performed for publication year, mean age of participants, type of cell (RPE, NSC, UMSC, and BMSC; ADSC was used as reference), and application of scaffold. Cochran’s *Q* test and *I*^2^ statistics were used to assess the heterogeneity, and a *p*-value < 0.1 was considered significant. Variances were evaluated at three levels: sampling variance (level 1), between effect sizes variance (level 2), and between-study variances (level 3). All meta-analyses were performed using ‘metafor’ and ‘meta’ packages (R programming language v 4.2.1).

## Results

### Study selection

**Fig. 1:**
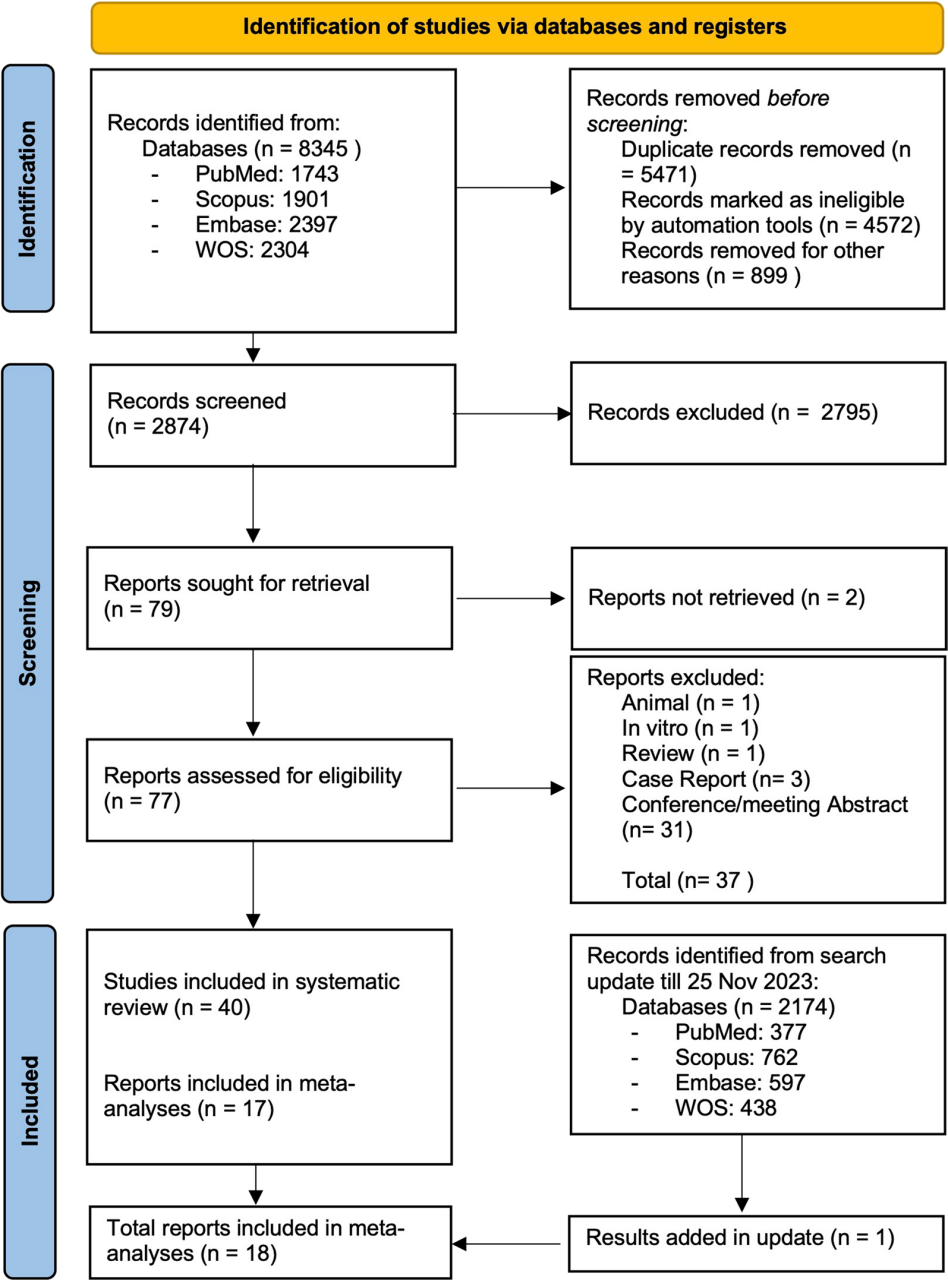
Flow diagram of literature search and study selection

After searching the databases, a total of 8345 records were retrieved. After removing duplicates, 2874 results remained for screening, of which 79 articles remained following title/abstract screening [23–101]. During the full-text screening process, 39 publications did not fulfill the eligibility requirements and were eliminated for various reasons, according to PRISMA guidelines [23–30, 34–38, 41, 47, 48, 50, 56, 59–62, 64, 71, 73, 74, 77, 79, 80, 83, 84, 86, 88, 89, 96–99, 102]. There were articles whose findings could not be included in this review due to being in vitro [59], in vivo [28], case reports [23, 74, 88], review article [79], conference/meeting abstracts [24–27, 29, 30, 34–38, 41, 47, 48, 50, 56, 60–62, 64, 71, 73, 77, 80, 83, 86, 96–99, 102], or full-text articles that could not be found [84, 89]. Based on the inclusion and exclusion criteria, 40 studies were considered for this study, of which 23 did not have enough data [40, 42, 45, 52–55, 58, 63, 67–69, 72, 75, 76, 78, 81, 91–94, 100, 101], and 17 had enough quantitative data to be included in the meta-analysis and presented in this study [33, 43, 46, 51, 65, 70, 85, 87, 103–111]. One article was added to the SMD meta-analysis by updating the search until November 25th [112]. The study selection process has been outlined in Fig. 1.

### Study characteristics and quality assessment

The included studies’ publication times were between 2006 and 2023. Out of the 18 studies that were reviewed, 12 studies have focused on patients diagnosed with AMD [33, 43, 46, 51, 65, 70, 85, 105, 106, 108, 109, 111], 7 studies were focused on patients with SMD [70, 85, 87, 105, 107, 110, 112], and a mere 2 studies were related to RP patients [103, 104]. In a total of 18 investigations, ESC was employed as a therapeutic approach in 9 studies [33, 43, 85, 87, 106–109, 112], while adult stem cells (ASCs) were used as a treatment in the remaining 9 trials [46, 51, 65, 70, 103–105, 110, 111]. Among the aforementioned studies, three investigations employed scaffold structures for the purpose of cultivating stem cells and facilitating therapeutic applications. In the study conducted by Kashani et al., a parylene membrane was applied as the scaffold [43, 108]. In addition, Da Cruz et al. employed a scaffold composed of a human-vitronectin-coated polyester membrane [33]. The summary of the characteristics of the studies is available in Table 1. The total number of the included eyes in the LogMAR meta-analysis is 224. Table 2 presents the NOS scoring of the included studies. All of the clinical trial studies included in the analysis received a score of ≥ 6 out of 9.

**Table 1:** Summary of findings from studies included in the meta-analysis

Author	Year	Condition	Number of eyes	Female percentage	Maximum follow-up	Dosage	Application method	Cells	Type of stem cell	Scaffold or cell suspension	Wet or dry AMD
Song [87]	2015	AMD	2	0%	52w	NA	Subretinal injection	RPE	ESC	Cell suspension	dry
Song [87]	2015	SMD	2	0%	52w	NA	Subretinal injection	RPE	ESC	Cell suspension	NA
Park [85]	2015	AMD	2	NA	6m	NA	Intravitreal injection	BMSC	ASC	Cell suspension	dry
Park [85]	2015	SMD	2	NA	6m	NA	Intravitreal injection	BMSC	ASC	Cell suspension	NA
Limoli [50]	2016	AMD	36	64%	6m	NA	Suprachoroidal injection	ADSC	ASC	Cell suspension	dry
Cotrim [110]	2017	AMD	10	NA	3m	NA	Intravitreal injection	BMSC	ASC	Cell suspension	dry
Kumar [107]	2017	AMD	30	37%	6m	NA	Intravitreal injection	BMSC	ASC	Cell suspension	dry
Da Cruz [109]	2018	AMD	2	50%	12m	NA	Subretinal implant	RPE	ESC	Cell sheets on human-vitronectin-coated polyester membrane	wet
Kashani [46]	2018	AMD	4	75%	over 6m	NA	Subretinal implant	RPE	ESC	Cell sheets on parylene membrane	dry
Liu [9]	2018	AMD	3	67%	12m	10^6/100ul	Subfoval injection	RPE	ESC	Cell suspension	wet
Oner [70]	2018	AMD	4	25%	6m	NA	Suprachoroidal	ADSC	ASC	Cell suspension	dry
Oner [70]	2018	SMD	4	50%	6m	NA	Suprachoroidal	ADSC	ASC	Cell suspension	NA
Heier [43]	2019	AMD	21	NA	12m	3*10^5/50ul	Subretinal injection	UMSC	ESC	Cell suspension	dry
Cotrim [33]	2020	SMD	10	60%	6m	NA	Intravitreal injection	BMSC	ASC	Cell suspension	NA
Nittala [105]	2020	AMD	11	18%	12m	NA	Subretinal injection	NSC	ASC	Cell suspension	dry
Sung [104]	2020	SMD	3	0%	36m	NA	Subretinal injection	RPE	ESC	Cell suspension	NA
Kashani [108]	2021	AMD	15	60%	12m	NA	Subretinal implant	RPE	ESC	Cell sheets on parylene membrane	dry
Li [106]	2021	SMD	7	100%	60m	NA	Subretinal injection	RPE	ESC	Cell suspension	NA
Tuekprakhon [103]	2021	RP	14	43%	12m	gp1, 10^6 gp2, 5*10^6 gp3, 10^7	Intravitreal injection	BMSC	ASC	Cell suspension	NA
Wiącek [102]	2021	RP	30	40%	12m	NA	Intravitreal injection	BMSC	ASC	Cell suspension	NA
Brant Fernandes [111]	2023	SMD	12	75%	12m	10^6/100ul	Subretinal injection	RPE	ESC	Cell suspension	NA

AMD, age-related macular degeneration; SMD, Stargardt’s macular dystrophy; RP, retinitis pigmentosa; RPE, retinal pigment epithelium; ESC, embryonic stem cell; NSC, neural stem cell; ASC, adult stem cell; BMSC, bone marrow mesenchymal stem cell; ADSC, adipose-derived mesenchymal stem cell; UMSC, umbilical cord mesenchymal stem cell

**Table 2:** Assessment of the quality of the included studies using the Joanna Briggs Institute’s (JBI) scale

Year	Author	Criteria and Corresponding Score									Overall
		#1	#2	#3	#4	#5	#6	#7	#8	#9	
2015	Song	1	1	1	1	1	1	1	1	NA	8
2015	Park	1	1	1	0	1	1	1	1	0	7
2016	Limoli	1	1	1	0	1	1	1	1	1	8
2017	Cotrim	1	1	1	0	1	0	1	1	0	6
2017	Kumar	1	1	1	1	1	1	1	1	1	9
2018	Da Cruz	1	1	1	0	1	1	1	1	1	8
2018	Kashani	1	1	1	1	1	0	1	1	NA	7
2018	Liu	1	1	1	1	1	1	1	1	NA	8
2018	Oner	1	1	1	1	1	1	1	1	NA	8
2019	Heier	1	1	1	1	1	0	1	1	NA	7
2020	Cotrim	1	1	1	1	1	1	1	1	1	9
2020	Nittala	1	1	0	1	1	1	1	1	1	8
2020	Sung	1	1	1	0	1	1	1	1	NA	7
2021	Kashani	1	0	1	0	1	1	1	1	NA	6
2021	Li	1	1	1	1	1	0	1	1	1	8
2021	Tuekprakhon	1	1	1	1	1	1	1	1	1	9
2021	Wiacek	1	1	1	1	1	1	1	1	1	9
2023	Brant Fernandes	1	1	1	0	1	1	1	1	1	8

#1 Is it clear in the study what is the ‘cause’ and what is the ‘effect’ (i.e. there is no confusion about which variable comes first)? #2 Were the participants included in any comparisons similar? #3 Were the participants included in any comparisons receiving similar treatment/care, other than the exposure or intervention of interest? #4 Was there a control group? #5 Were there multiple measurements of the outcome both pre and post the intervention/exposure? #6 Was follow up complete and if not, were differences between groups in terms of their follow up adequately described and analyzed? #7 Were the outcomes of participants included in any comparisons measured in the same way? #8 Were outcomes measured in a reliable way? #9 Was appropriate statistical analysis used? *NA* not applicable/unclear

### Cell therapy for age-related macular degeneration

**Fig. 2:**
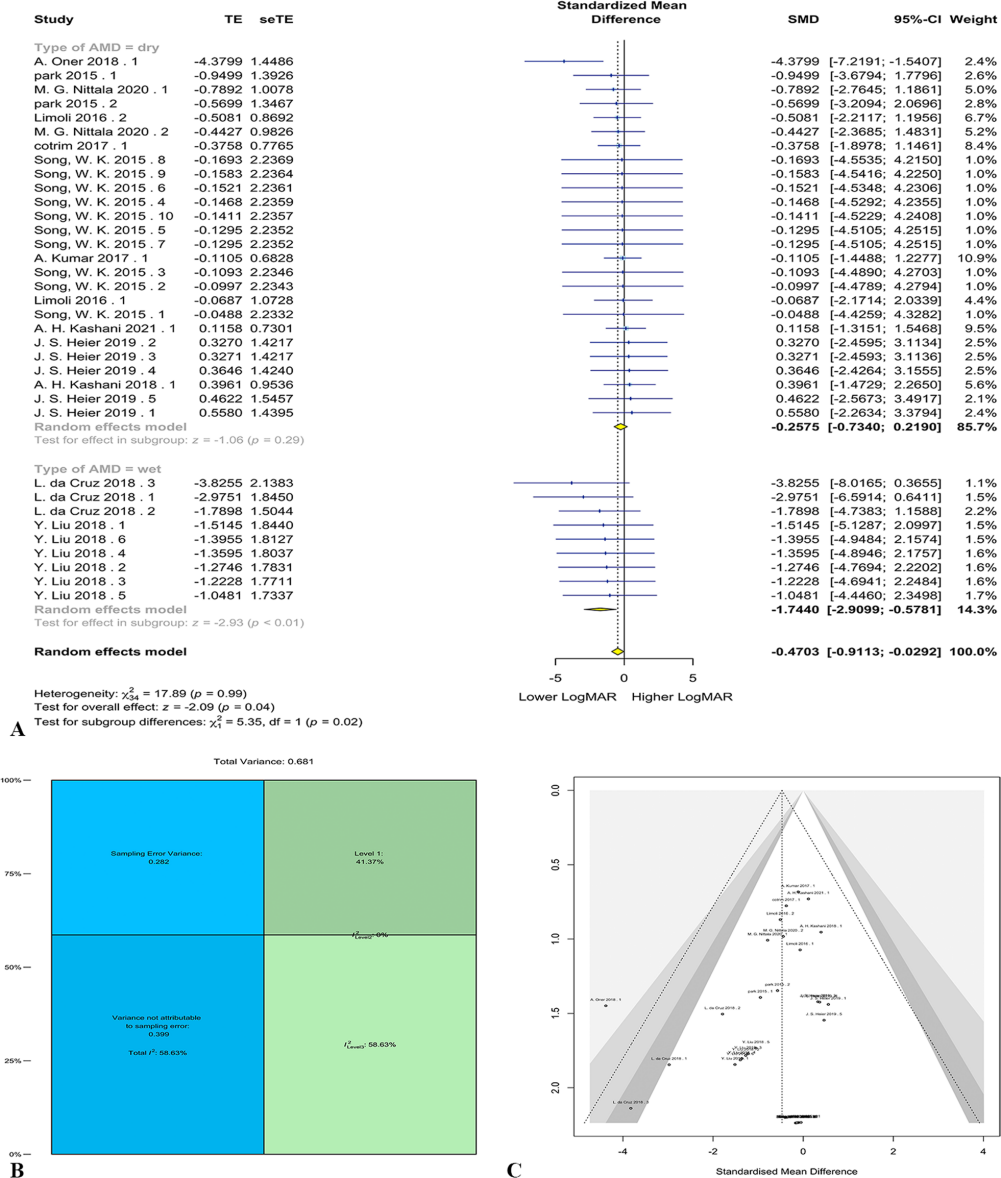
AMD meta-analysis results. Forest plot for AMD meta-analysis (**A**). Distribution of variances in different levels of analysis (**B**). Funnel plot for publication bias of the included studies (**C**)

Overall, 12 studies were included in the meta-analysis, through which 140 eyes underwent the cell therapy intervention for AMD. The random-effect three-level meta-analysis demonstrated that cell therapy decreased the LogMAR score compared to baseline (g = − 0.47, 95% CI = − 0.91 to − 0.03, *p* = 0.04). The forest plot for meta-analysis has been shown in Fig. 2A. Heterogeneity was significant (*Q* = 52.51, *p* < 0.05) with the heterogeneity variance components of τ_level 3_ = 0.40 and τ_level 2_ = 0.00. Also, it was demonstrated that 58.63% of heterogeneity was attributable to level 3 (${I}_{level 3}^{2}$; between-cluster variance), 0% was attributable to level2 (${I}_{level 2}^{2}$; within-cluster variance), and 41.37% was due to sampling error variance (level 1). Variance components have been shown in Fig. 2B. The three-level model was shown to be superior to the two-level model, where within-study variance is disregarded based on the likelihood ratio test (*X*^2^ = 15.73, *p* < 0.0001).

Subgroup analysis was conducted to determine whether the type of AMD, wet or dry, has an impact on the results. It was observed that the decrease in the LogMAR was significant in the “wet” AMD (g = − 1.74, 95% CI = − 2.91 to − 0.58, *p* < 0.01), but not in “dry” AMD (g = − 0.26, 95% CI = − 0.73–0.22, *p* = 0.29). The heterogeneity for wet and dry subgroups was not significant (both *p*-values = 0.99), indicating that the heterogeneity was primarily due to the type of AMD. The chi-squared test for evaluating disparity between subgroups was significant (*X*^2^ = 5.35, *p* < 0.05).

Egger’s linear regression test for funnel plot asymmetry was insignificant, demonstrating no substantial publication bias (*p* = 0.09; Fig. 2C). Sensitivity analysis demonstrated that “A. Oner 2018” [105] analysis woutlier. Repeating the analysis without “A. Oner 2018” does not change the overall significance of the analysis (*p* = 0.10). Moreover, the significance of “wet” and “dry” subgroups and the difference between subgroups does not change (*p* < 0.01, *p* = 0.58, and *p* < 0.05, respectively). Meta-regression demonstrated no significant influence on bone marrow mesenchymal stem cell (BMSC) and umbilical cord MSC (UMSC), with *p*-values of 0.17 and 0.11. However, it was observed that NSC, RPE, publication year, the mean age of participants, and application of scaffold have significant effects on the overall LogMAR (*p* < 0.05, *p* < 0.001, *p* < 0.05, *p* < 0.0001, and *p* < 0.01, respectively).

### Cell therapy for stargardt macular degeneration

**Fig. 3:**
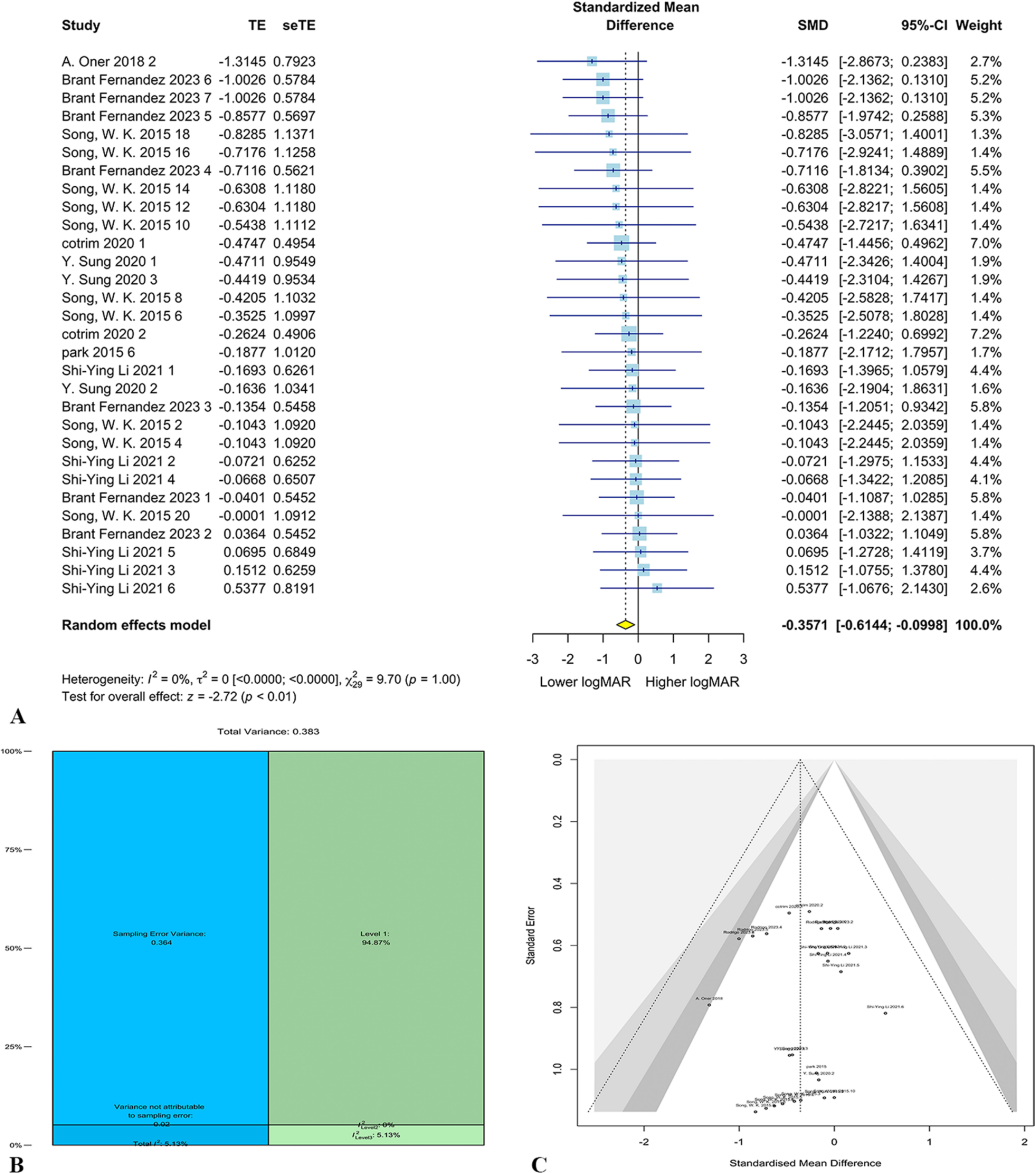
SMD meta-analysis results. Forest plot for SMD meta-analysis (**A**). Distribution of variances in different levels of analysis (**B**). Funnel plot for publication bias of the included studies (**C**)

Overall, 7 studies with 40 eyes entered the meta-analysis for cell therapy in SMD. Through random-effect Three-level meta-analysis, it was observed that cell therapy significantly reduced the LogMAR score (g = − 0.36, 95% CI = − 0.61–0.01, *p* < 0.01). The forest plot for meta-analysis has been shown in Fig. 3A. The heterogeneity was not significant (*Q* = 9.70, *p* = 1.00) with heterogeneity variance components of τ_level 3_ = 0.02 and τ_level 2_ = 0.00. Also, it was demonstrated that 5.13% of heterogeneity was attributable to level 3 (${I}_{level 3}^{2}$), 0% was attributable to level 2 (${I}_{level 2}^{2}$), and 94.87% was because of sampling error variance (level 1). Variance components have been shown in Fig. 3B. The likelihood ratio test demonstrated the three-level model does not provide a significantly better fit compared to the two-level model (*X*^2^ = 0.26, *p* = 0.61). The publication bias was not significant based on Egger’s linear regression (*p* = 0.96; Fig. 3C). No potential outlier was detected in the sensitivity analysis. The meta-regression demonstrated no significant effect for publication year, BMSC, and RPE with *p*-values of 0.74, 0.15, and 0.09. However, the mean age of participants has a significant effect on the overall LogMAR (*p* < 0.05).

### Cell therapy for retinitis pigmentosa

**Fig. 4:**
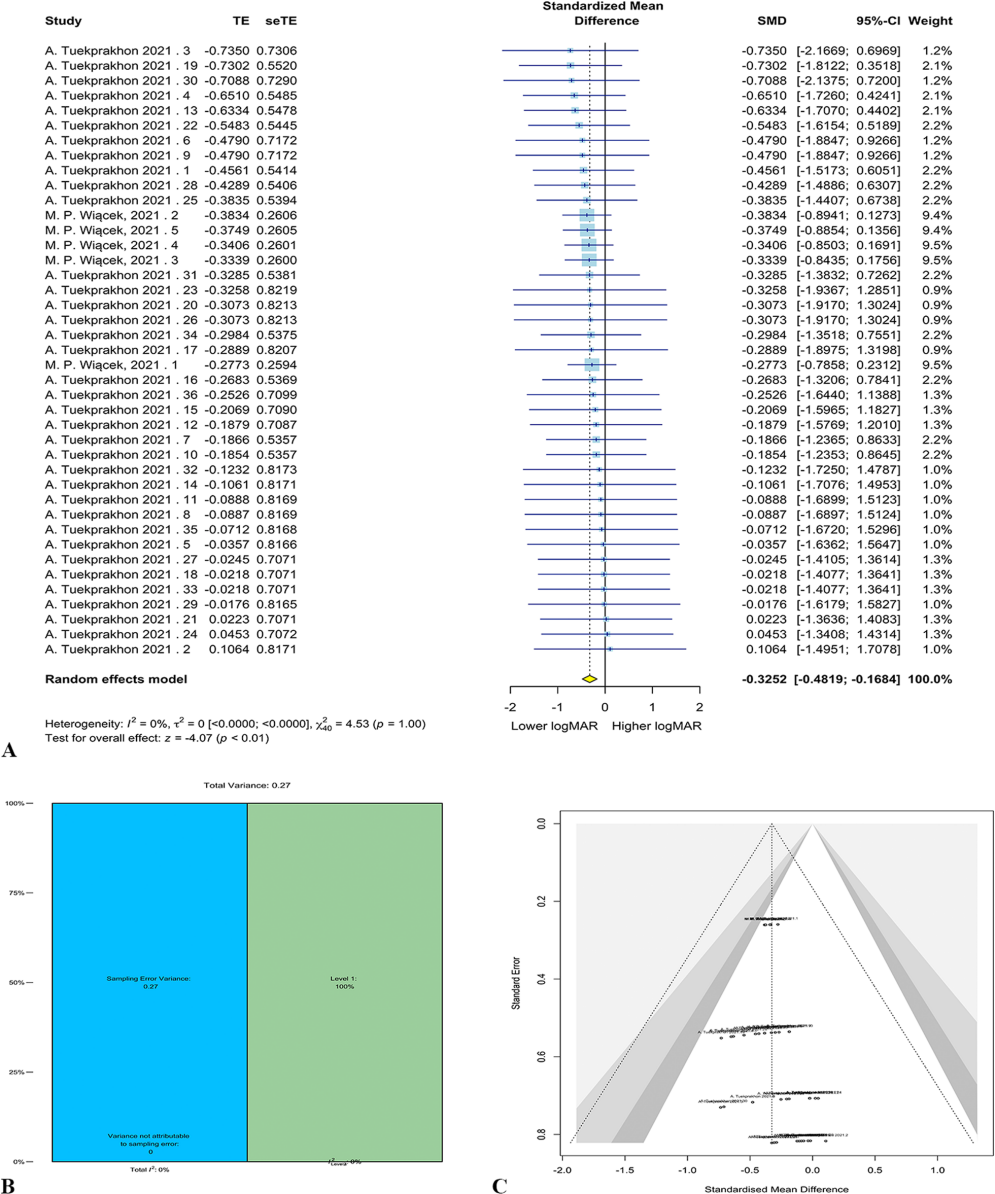
RP meta-analysis results. Forest plot for RP meta-analysis (**A**). Distribution of variances in different levels of analysis (**B**). Funnel plot for publication bias of the included studies (**C**)

Only 2 studies with 44 eyes were included in the meta-analysis. A significant improvement was achieved by cell therapy based on the three-level random-effect meta-analysis results (g = − 0.33, 95% CI = − 0.48 to − 0.17, *p* < 0.0001). The forest plot for meta-analysis has been shown in Fig. 4A. No significant heterogeneity was observed (*Q* = 4.53, *p* = 1.00). The variance components are τ_level 3_ = 0.00 and τ_level 2_ = 0.00. All heterogeneity was attributable to level 1 (${I}_{level 1}^{2}$ = 100%; Fig. 4B). Also, the likelihood ratio test demonstrated that the three-level model was not superior to the two-level model (*X*^2^ = 0.00, *p* = 1.00). The Egger’s test showed no significant publication bias (*p* = 0.09; Fig. 4C). Sensitivity analysis revealed no outlier. The meta-regression was not performed since the effects were only extracted from two studies and the results may not be reliable.

## Discussion

In this meta-analysis, we assessed the effect of cell-based therapies on patients with different types of retinal degeneration, with a specific focus on Age-related Macular Degeneration (AMD), Stargardt Macular Degeneration (SMD), and Retinitis Pigmentosa (RP). Our results were in line with prior studies that affirmed the efficacy of stem cell transplantation as an effective and safe therapeutic modality for individuals diagnosed with RDDs.

In this study, encompassing 18 studies and a total of 224 eyes, we quantitively assessed the improvement of best-corrected visual acuity among patients with AMD, SMD, or RP who have undergone stem cell therapy. Cell therapy resulted in decreased LogMAR scores for both types of AMD, whereas the improvement was only significant in wet-type AMD (*p* < 0.01) but not dry-type AMD (*p* = 0.29). Moreover, the utilization of cell therapy improved the visual acuity in SMD and RP (*p* < 0.01 and *p* < 0.0001, respectively). The study findings have been summarized in Table 3. Occurrence of severe adverse events following stem cell therapy was infrequent, and most of the participants experienced only a mild ocular side effect. To the best of our knowledge, this is the first available meta-analysis to examine the effect of cell therapy in all three prevalent retinal degenerative disorders concurrently. Most of the available previous studies have investigated cell-based therapy in one or two particular types of disorders [113–115]. Our study has included both types of AMD, RP, and SMD all together in one comprehensive study.

**Table 3:** Summary of findings from studies included in the meta-analysis and meta-regression

Study	Subgroup	No. Studies	No. eyes	Meta-analysis				Meta-regression		
				Standardized MD	95%CI	P-value	Subgroup difference	Variable	estimate	P-value
AMD	Wet	2	5	− 1.74	− 2.91 to − 0.58	< 0.01	< 0.05	Publication year	0.35	< 0.05
								Mean age	0.30	< 0.0001
	Dry	10	135	− 0.26	− 0.73–0.22	< 0.01		Scaffold	− 3.50	< 0.001
								RPE	1.90	< 0.0001
								NSC	− 1.97	< 0.05
	Overall	12	140	− 0.47	− 0.91 to − 0.03	< 0.05				
								BMSC	0.50	0.17
								UPMSC	− 1.16	0.11
SMD	Overall	2	40	0.25	− 0.52–1.02	0.52	NA	Publication year	− 0.01	0.74
								Mean age	− 0.03	< 0.05
								RPE	1.38	0.09
								BMSC	1.24	0.15
RP	Overall	2	44	0.41	0.16–0.67	< 0.01	NA	NA	NA	NA

MD, mean difference; AMD, age-related macular degeneration; SMD, Stargardt’s macular dystrophy; RP, retinitis pigmentosa; RPE, retinal pigment epithelium; ESC, embryonic stem cell; NSC, neural stem cell; ASC, adult stem cell; BMSC, bone marrow mesenchymal stem cell; ADSC, adipose-derived mesenchymal stem cell; UMSC, umbilical cord mesenchymal stem cell

We presented queries such as “What impact does stem cell therapy have on visual performance in patients with retinal degenerative disorders?” and “Which factors can modify this impact?” across a range of clinical trial studies. Accordingly, it became imperative to carry out this meta-analysis for common retinal degenerative disorders to address these questions.

Cells used in the studies were either differentiated or undifferentiated stem cells. The stem cells that have been used are BMSC, UMSC, NSC, and ADSC. The stem cells underwent quality control, safety, and purity evaluation before application. RPEs are commonly obtained through spontaneous differentiation from specific human embryonic stem cell lines. Various methods were used to characterize RPE, confirm their purity, and avoid the inclusion of undifferentiated cells, including immunocytochemistry, fluorescence-activated cell sorting (FACS), electron microscopy, genetic analysis, and polymerase chain reaction (PCR) [33, 43, 65, 85, 103, 116].

In a study led by Takahashi et al., the effect of RPE cells derived from iPSC transplantation was assessed in right eye of a female patient with wet AMD. A four-year follow-up revealed that stem cells had survived and maintained a normal morphology. Although this study did not result in improved vision, the patient’s vision remained stable after the intervention despite a continuous reduction in the previous years [88]. In our study, A subgroup analysis based on AMD types revealed an increase in visual acuity following stem cell therapy in both types of AMD; however, LogMAR reduction was statistically significant only in patients with wet AMD. Da Cruz et al. developed an RPE patch comprised of hESC-derived RPE on an artificial basement membrane and implanted it into the subretinal space of two patients with severe exudative AMD. Based on their results, visual acuity was improved in both patients, gaining 29 and 21 letters over the course of 12 months of follow-up [33]. Additionally, a metanalysis has shown that stem cell transplantation would significantly improve visual acuity in dry-type AMD patients in 6 and 12-month follow-ups [113].

Our results align with prior studies providing evidence that cell transplantation is a potentially effective and safe treatment option for individuals diagnosed with RP or SMD [117–119]. Huang et al. reviewed 404 eyes with RP and 92 with SMD. BCVA improved significantly in both RP and SMD groups in 6-month follow-ups with a reduction of LogMAR score of − 0.12 and − 0.14 in each group, respectively. The outcome of cell therapy in RP patients in 12-month follow-ups showed a marginal yet significant improvement in visual acuity at the 12-month assessment point [118].

Stem cells are capable of renewing themselves through cell division and can differentiate into multi-lineage cells [120]. These cells are categorized as embryonic stem cells, induced pluripotent stem cells, and adult stem cells, particularly mesenchymal stem cells [13]. Several experimental studies have shown that transplanted stem cells can survive and enhance the functionality of damaged cells in degenerated retina [121–123]. Increased expression of retinal markers [124], prolongation of photoreceptors survival [125], reduction of retinal cell apoptosis [126], and improved visual outcomes [127, 128] have been detected following stem cell transplantation in animal models. Furthermore, intravitreal injection of stem cells has led to a reduction of inflammation markers and retinal damage [129]. A mitigating effect on oxygen-induced retinal damage in mouse models has also been seen following the utilization of endothelial cells derived from human-induced pluripotent stem cells, which has led to a reduction in pathological vaso-occlusion and neovascularization [130].

Finally, it should be noted that the number of studies included in the meta-analysis was limited, particularly with regard to RP and SMD. This could potentially impact the final results of the analysis and introduce bias. Additionally, the genetic characteristics and variability of the patients were only reported in a few studies and could not be taken into account in the meta-analysis as a possible factor influencing the results. Furthermore, due to inconsistencies in the reporting of outcome measures beyond visual acuity and the limited availability of studies providing data on these outcomes, it was not possible to include them in the meta-analysis. Therefore, further research should consider these factors for a more comprehensive understanding of the topic.

## Conclusion

In summary, stem cell therapy has also been seen to be a potential treatment modality for retinal degeneration disorders, enhancing the visual acuity of those affected. Nevertheless, more studies and clear guidelines are needed to corroborate initial results. Future research should focus on acknowledging stem cell therapy mechanisms and comparing various stem cell types for efficacy. Our study had some limitations. First, we only used BCVA to compare the overall effect of stem cell therapy, and results from spectral domain-optical coherence tomography (SD-OCT), ERG, and fundus autofluorescence were not applied in this study. Second, we did not include post-therapeutic adverse effects, and hence, future studies are needed to compare cell therapy safety in various routes of stem cell transplantation. Third, we could not investigate the long-term impact given the data shortage.

### Supplementary Information

**Supplementary file 1: Comprehensive list of search strategies for all databases.**

**Supplementary file 2: Comprehensive list of search strategies for all databases.**

## Data Availability

The datasets used and/or analyzed during the current study are available from the corresponding author upon reasonable request.
