# Supplementary material for: Cell therapy for retinal degenerative disorders: a systematic review and three-level meta-analysis
Source: J Transl Med. 2024 Mar 2;22:227. doi: 10.1186/s12967-024-05016-x (PMC10908175; doi:10.1186/s12967-024-05016-x)
Supplement: Supplementary file 1 — Comprehensive list of search strategies for all databases. [file 12967_2024_5016_MOESM1_ESM.docx]

WOS = Results: 2,809 11/23/2021

TOPIC: (“macular degeneration” OR “Geographic Atrophy” OR “Macular Edema” OR “Stargardt Disease” OR “Vitelliform Macular Dystrophy” OR “wet macular degeneration” OR “Retinal Drusen” OR “retinal dystrophies” OR “Cone-Rod Dystrophies” OR “Cone Rod Dystrophies” OR “Retinitis Pigmentosa” OR Retinoschisis OR “Alstrom Syndrome” OR “Usher Syndromes” OR “age-related macular degeneration” OR “age related macular degeneration” OR “Retinal degeneration” OR “Stargardt's macular dystrophy” OR “Stargardt's macular degeneration” OR “Macular dystrophy” OR “Age-related maculopathy” OR “Dry macular degeneration” OR “Tapetoretinal degeneration” OR “Juvenile macular degeneration”) AND TOPIC: (“Stem cell” OR “Stem cells” OR “human embryonic stem cell*” OR hESCs OR “induced pluripotent stem cell*” OR iPSCs OR “human umbilical tissue-derived cell*” OR “Mesenchymal Stem Cell*” OR MSCs OR “retinal progenitor cell*” OR RPCs OR “Cell- and Tissue-Based Therapy” OR “Cell Therapy” OR “regenerative medicine” OR “Tissue engineering” OR Scaffold OR Scaffolds OR “tissue engineered Construct” OR “Extracellular matrix” OR “Acellular Scaffold” OR Decellularization OR Recellularization)

Update till 11/25/2023

438 results

TS=(“macular degeneration” OR “Geographic Atrophy” OR “Macular Edema” OR “Stargardt Disease” OR “Vitelliform Macular Dystrophy” OR “wet macular degeneration” OR “Retinal Drusen” OR “retinal dystrophies” OR “Cone-Rod Dystrophies” OR “Cone Rod Dystrophies” OR “Retinitis Pigmentosa” OR Retinoschisis OR “Alstrom Syndrome” OR “Usher Syndromes” OR “age-related macular degeneration” OR “age related macular degeneration” OR “Retinal degeneration” OR “Stargardt's macular dystrophy” OR “Stargardt's macular degeneration” OR “Macular dystrophy” OR “Age-related maculopathy” OR “Dry macular degeneration” OR “Tapetoretinal degeneration” OR “Juvenile macular degeneration”) AND TS=(“Stem cell” OR “Stem cells” OR “human embryonic stem cell*” OR hESCs OR “induced pluripotent stem cell*” OR iPSCs OR “human umbilical tissue-derived cell*” OR “Mesenchymal Stem Cell*” OR MSCs OR “retinal progenitor cell*” OR RPCs OR “Cell- and Tissue-Based Therapy” OR “Cell Therapy” OR “regenerative medicine” OR “Tissue engineering” OR Scaffold OR Scaffolds OR “tissue engineered Construct” OR “Extracellular matrix” OR “Acellular Scaffold” OR Decellularization OR Recellularization)

PubMed = Results: 1,743 11/23/2021

377 update till 25 Nov 2023

("macular degeneration"[Title/Abstract] OR "Geographic Atrophy"[Title/Abstract] OR "Macular Edema"[Title/Abstract] OR "Stargardt Disease"[Title/Abstract] OR "Vitelliform Macular Dystrophy"[Title/Abstract] OR "wet macular degeneration"[Title/Abstract] OR "Retinal Drusen"[Title/Abstract] OR "retinal dystrophies"[Title/Abstract] OR "Cone-Rod Dystrophies"[Title/Abstract] OR "Cone Rod Dystrophies"[Title/Abstract] OR "Retinitis Pigmentosa"[Title/Abstract] OR Retinoschisis[Title/Abstract] OR "Alstrom Syndrome"[Title/Abstract] OR "Usher Syndromes"[Title/Abstract] OR "age-related macular degeneration"[Title/Abstract] OR "age related macular degeneration"[Title/Abstract] OR "Retinal degeneration"[Title/Abstract] OR "Stargardt's macular dystrophy"[Title/Abstract] OR "Stargardt's macular degeneration"[Title/Abstract] OR "Macular dystrophy"[Title/Abstract] OR "Age-related maculopathy"[Title/Abstract] OR "Dry macular degeneration"[Title/Abstract] OR "Tapetoretinal degeneration"[Title/Abstract] OR "Juvenile macular degeneration"[Title/Abstract]) AND ("Stem cell"[Title/Abstract] OR "Stem cells"[Title/Abstract] OR "human embryonic stem cell*"[Title/Abstract] OR hESCs[Title/Abstract] OR "induced pluripotent stem cell*"[Title/Abstract] OR iPSCs[Title/Abstract] OR "human umbilical tissue-derived cell*"[Title/Abstract] OR "Mesenchymal Stem Cell*"[Title/Abstract] OR MSCs[Title/Abstract] OR "retinal progenitor cell*"[Title/Abstract] OR RPCs[Title/Abstract] OR "Cell-Based Therapy"[Title/Abstract] OR "Tissue-Based Therapy"[Title/Abstract] OR "Cell Therapy"[Title/Abstract] OR "regenerative medicine"[Title/Abstract] OR "Tissue engineering"[Title/Abstract] OR Scaffold[Title/Abstract] OR Scaffolds[Title/Abstract] OR "tissue engineered Construct"[Title/Abstract] OR "Extracellular matrix"[Title/Abstract] OR "Acellular Scaffold"[Title/Abstract] OR Decellularization[Title/Abstract] OR Recellularization[Title/Abstract])

Scopus = Results: 3,893

Update till 25 nov 2023 🡪 762

( TITLE-ABS-KEY ( "macular degeneration" OR "Geographic Atrophy" OR "Macular Edema" OR "Stargardt Disease" OR "Vitelliform Macular Dystrophy" OR "wet macular degeneration" OR "Retinal Drusen" OR "retinal dystrophies" OR "Cone-Rod Dystrophies" OR "Cone Rod Dystrophies" OR "Retinitis Pigmentosa" OR retinoschisis OR "Alstrom Syndrome" OR "Usher Syndromes" OR "age-related macular degeneration" OR "age related macular degeneration" OR "Retinal degeneration" OR "Stargardt's macular dystrophy" OR "Stargardt's macular degeneration" OR "Macular dystrophy" OR "Age-related maculopathy" OR "Dry macular degeneration" OR "Tapetoretinal degeneration" OR "Juvenile macular degeneration" ) AND TITLE-ABS-KEY ( "Stem cell*" OR "Human Embryonic Stem Cells" OR "human embryonic stem cell*" OR "HESCs" OR "Induced Pluripotent Stem Cells" OR "induced pluripotent stem cell*" OR "Fibroblast-Derived Induced Pluripotent Stem Cell*" OR "IPSCs" OR "human umbilical tissue-derived cell*" OR "Mesenchymal Stem Cell*" OR "MSCs" OR "retinal progenitor cell*" OR "RPSCs" OR "Cell- and Tissue-Based Therapy" OR "Cell Therapy" OR "regenerative medicine" OR "Tissue engineering" OR scaffold OR scaffolds OR construct OR "Extracellular matrix" OR "Acellular Scaffold" OR "decellularization" OR "recellularization" ) )

Embase

Update till nov 25 2023 🡪 597

('macular degeneration':ti,ab OR 'geographic atrophy':ti,ab OR 'macular edema':ti,ab OR 'stargardt disease':ti,ab OR 'vitelliform macular dystrophy':ti,ab OR 'wet macular degeneration':ti,ab OR 'retinal drusen':ti,ab OR 'retinal dystrophies':ti,ab OR 'cone-rod dystrophies':ti,ab OR 'cone rod dystrophies':ti,ab OR 'retinitis pigmentosa':ti,ab OR 'retinoschisis':ti,ab OR 'alstrom syndrome':ti,ab OR 'usher syndromes':ti,ab OR 'age-related macular degeneration':ti,ab OR 'age related macular degeneration':ti,ab OR 'retinal degeneration':ti,ab OR 'stargardts macular dystrophy':ti,ab OR 'stargardtsmacular degeneration':ti,ab OR 'macular dystrophy':ti,ab OR 'age-related maculopathy':ti,ab OR 'dry macular degeneration':ti,ab OR 'tapetoretinaldegeneration':ti,ab OR 'juvenile macular degeneration') AND ('stem cell':ti,ab OR 'stem cells':ti,ab OR 'human embryonic stem cell*':ti,ab OR 'hescs':ti,ab OR 'induced pluripotent stem cell*':ti,ab OR 'ipscs':ti,ab OR 'human umbilical tissue-derived cell*':ti,ab OR 'mesenchymal stem cell*':ti,ab OR 'mscs':ti,ab OR 'retinal progenitor cell*':ti,ab OR 'rpcs':ti,ab OR 'cell- and tissue-based therapy':ti,ab OR 'cell therapy':ti,ab OR 'regenerative medicine':ti,ab OR 'tissue engineering':ti,ab OR 'scaffold':ti,ab OR 'scaffolds':ti,ab OR 'tissue engineered construct':ti,ab OR 'extracellular matrix':ti,ab OR 'acellular scaffold':ti,ab OR 'decellularization':ti,ab OR 'recellularization':ti,ab)
